# Effect of L-arginine on HSP70 expression in liver in weanling piglets

**DOI:** 10.1186/1746-6148-9-63

**Published:** 2013-04-04

**Authors:** Xin Wu, Chunyan Xie, Yulong Yin, Fengna Li, Tiejun Li, Ruilin Huang, Zheng Ruan, Zeyuan Deng

**Affiliations:** 1 Scientific Observing and Experimental Station of Animal Nutrition and Feed Science in South-Central, Ministry of Agriculture, Hunan Provincial Engineering Research Center of Healthy Livestock, Key Laboratory of Agro-ecological Processes in Subtropical Region, Institute of Subtropical Agriculture, Chinese Academy of Sciences, Changsha, Hunan, 410125, China; 2 State Key Laboratory of Food Science and Technology and College, Nanchang University, Nanchang, 330031, China; 3 Institute of Subtropical Agriculture, Chinese Academy of Sciences, Changsha, Hunan, 410125, China

**Keywords:** L-arginine, Liver, HSP70, Weanling piglets

## Abstract

**Background:**

This study was conducted to evaluate the effects of L-arginine (Arg) on photomicrographs and HSP70 expression in the liver of weanling piglets. Twelve healthy Landrace × Yorkshire piglets that had been weaned at 21 d (average body weight 5.56 ± 0.51 kg) were randomly divided into a control group and an Arg group (6 g/kg feed). At age 28 d, all of the piglets were slaughtered to obtain liver samples to determine HSP70 expression by real-time PCR, western blot and immunohistochemistry.

**Results:**

The results showed that, compared to control piglets, treatment with Arg decreased inflammatory reactions caused by weaning. The immunohistochemical localization of HSP70 in liver revealed strong expression in the Arg group. Arg increased HSP70 mRNA and HSP70 expression in the liver (P < 0.05).

**Conclusions:**

These findings suggest that dietary supplementation with Arg could maintain liver health by inducing HSP70 expression in weanling piglets.

## Background

As the largest internal organ, the liver is responsible for about 500 vital bodily functions, and plays important roles in digestion and metabolism by regulating the production, storage, and release of sugar, fats, and cholesterol. The liver produces a variety of important proteins, including enzymes, hormones, blood proteins, clotting factors, and immune factors. As the main site of detoxification and the primary defense barrier, the liver is liable to be injured by weaning stress [1]. Weaning stress in piglets is often associated with reduced food consumption as well as temporary reductions in weight gain, which can result in postweaning lag, a period of decreased feed intake and increased diarrhea, disease, intestinal dysfunction and atrophy, and mortality in piglets [2-6]. A healthy liver can produce approximately 80 percent of the body’s required amino acids, among which L-arginine (Arg) is essential; when Arg turnover increases, as in growth, inflammation, or tissue repair, the dietary supply can become rate-limiting for Arg-metabolizing pathways [7-10]. Arg deficiency can result in hyperammonemia, and intestinal and immunological dysfunction [11,12]. The administration of Arg reduces damage to liver tissue after ischaemia injury [13]. Urea synthesis from primary hepatocytes seems to be a valid viability indicator, since mitochondrial transmembrane transport of the urea cycle intermediates ornithine and citrulline is involved in the urea cycle [14].

Heat shock protein 70 (HSP70) is a member of a highly conserved family that possess a variety of functions, but are best known for chaperoning and re-folding partially denatured protein [15-17]. The hepatic response to stress may involve the synthesis and accumulation of HSP70s [18,19]. Experimental evidence suggests that some amino acids such as glutamine and arginine regulate HSP expression, which is essential for preventing organ dysfunction [20]. The aim of the present study was to investigate the effects of Arg on photomicrographs and HSP70 expression in the liver under weaning stress in piglets.

## Methods

### Animals, housing and treatments

Twelve healthy Landrace × Yorkshire piglets weaned at 21 d (average weight 5.56 ± 0.51 kg) were randomly divided into a control group and an Arg group (6 g/kg feed). The diet in the control group was made iso-nitrogenous with the addition of appropriate amounts of alanine (12.3 g/kg feed) at the expense of lactose and glucose, as described by Kim et al. [21]. The nutrients in the basal diet were adequate for piglets to meet the NRC-recommended requirements within the weight range used in the present study [22]. The main ingredients in the basal diet were corn (51.55 g/kg), soybean meal (242 g/kg), fishmeal (60 g/kg), whey powder (90 g/kg), and cream (EE 50%, 60 g/kg) (Table 1). The diets were balanced with respect to the standardized content of DE (14.2 MJ/kg), CP (200 g/kg), true ileal digestible limiting amino acids (Lys, Met + Cys, Thr, Trp), Ca and P, according to our previous study [23]. The diets were administered three times daily at 08:00, 12:00, and 18:00, respectively. All of the animals had free access to drinking water.

**Table 1:** Composition of the basal diet for weanling pigs (as-fed basis)

	**Control group**	**Arg group**
Ingredients (%)		
Corn (8% CP; < 13% H_2_O)	49.9	49.9
Soybean expanded (43% CP)	24.2	24.2
Fish meal (CP 65%)	6.0	6.0
Whey (100%)	9.0	9.0
Cream powder (50% fat)	6.0	6.0
Limestone	0.5	0.5
Monocalcium phosphate	1.0	1.0
NaCl	0.2	0.2
Flavor	0.06	0.06
Mineral-vitamin premix^1^	1.0	1.0
L-Lysine · HCl	0.31	0.31
L-Methionine	0.06	0.06
L-Threonine	0.12	0.12
Glucose	0.42	1.05
L-alanine	1.23	0.0
L-Arginine	0.0	0.6
Total	100	100
Nutrient levels ^2^		
DE (MJ/kg)	14.21	14.21
Crude protein (%)	20.0	20.0
Calcium (%)	0.71	0.71
Available phosphorus (%)	0.48	0.48
L-Lysine (%)	1.35	1.35
L-Methionine (%)	0.39	0.39
L-Threonine (%)	0.91	0.91
L-Arginine (%)	0.49	1.09

^1^ Providing the following per kg diet: CuSO_4_ · 5H_2_O,19.8 mg; KI, 0.20 mg;FeSO_4_ · 7H_2_O, 400 mg; NaSeO_3_, 0.56 mg; ZnSO_4_ · 7H_2_O, 359 mg; MnSO_4_ · H_2_O, 10.2 mg; vitamin K (menadione), 5 mg; vitamin B_1_, 2 mg; vitamin B_2_, 15 mg; vitamin B_12_, 30 μg; vitamin A, 5400 IU; vitamin D_3_, 110 IU; vitamin E, 18 IU; choline chloride, 80 mg; antioxidants, 20 mg; Fungicide 100 mg.

^2^ Chemical composition was calcualted and not analyzed.

Arg was obtained from Ajinomoto (Tokyo, Japan).

The experiment was carried out in accordance with the Chinese Guidelines for Animal Welfare and approved by the Animal Welfare Committee of the Institute of Subtropical Agriculture, the Chinese Academy of Sciences.

### Sampling

At age 28 d, blood samples were obtained for the analysis of serum indices. All of the experimental piglets were anaesthetised with an i.v injection of sodium pentobarbital (50 mg/kg BW) and bled by exsanguination. Liver samples were rapidly frozen in liquid nitrogen and stored at −80 for real-time PCR and Western blotting analysis. Additional liver samples from the same location were fixed in 4% paraformaldehyde for two hours at 4, transferred to PBS overnight and 30% sucrose for 2 hours at 4, and then embedded in paraffin, sectioned, and stained with hematoxylin and eosin for histologic studies. The histological activity index (HAI) were evaluated according to Ishak and colleagues [24].

### Serum biochemical indices

An Automated Biochemistry Analyzer (Synchron CX Pro, Beckman Coulter, Fullerton, CA, USA) was used to determine the concentrations of serum glucose, glutamic-oxalacetic transaminase (GOT), glutamic-pyruvic transaminase (GPT), lactic acid (LAC)**,** lactate dehydrogenase (LDH), and alkaline phosphatase (ALP) activities, according to commercial kits and the manufacturer’s instructions. All kits were purchased from Beijing Chemlin Biotech Co., Ltd (Beijing, China).

Serum cortisol (COR) was determined by radioimmunoassay according to the instructions of the manufacturer of the corresponding reagent kit (China Institute of Atomic Energy, Beijing, China).

### Immunohistochemistry for HSP70

Paraffin-embedded liver tissue samples were processed using an immunohistochemical technique with specific anti-HSP70 (SPA-810 Stressgen Bioreagents, British Columbia, Canada) diluted 1:400 using PBS buffer. Samples were subjected to antigen retrieval and the reduction of non-specific binding. The tissue sections were incubated with primary antibody in a humidified chamber at 4°C. After the tissue sections were rinsed with Tris buffered saline (TBS), pH 7.4, they were incubated at room temperature for 30 min for the detection of HSP70. The BCIP-NBT substrate system (Sigma, St. Louis, MO) was used to detect alkaline phosphatase conjugate activity. The sections were then counterstained with haematoxylin for 5 s, and then rinsed, dried and mounted with coverslips. The sections were examined by light microscopy and images were captured with a digital camera.

### Real-time PCR for HSP70

Total RNA was extracted from liver tissue by a guanidinium isothiocyanate method using TrizolTM reagent (Gibco BRL, Berlin, Germany) and treated with DNase according to the manufacturer’s instructions. The mRNA of HSP70 was determined by a standard real-time polymerase chain reaction (PCR) method as previously described [25]. The following primer pairs were used: GAPDH: (Fwd—5’-GTCGGTGTGAACGGATTT-3’; Rev—5’-ACTCCACGACGTACTCAGC-3’) and HSP70 (Fwd—5’-GCCCTGAATCCGCAGAATA-3’; Rev—5’-TCCCCACGGTAGGAAACG-3’) based on the following conditions: 30 s of denaturation at 94, 30 s of annealing at 56, and 30 s of extension at 72 for 30 cycles.

### Western blot for HSP70

To confirm the results of immunohistochemistry, the expression of HSP70 in the liver was detected by Western blot assay. Western blot analysis was performed as described previously [26], with GAPDH as a loading control. One hundred-microgram samples were homogenized in 6 volumes of buffer A (20 mM HEPES, pH 7.4, 100 mM KCl, 0.2 mM EDTA, 2 mM EGTA, 1 mM DTT, 50 mM NaF, 50 mM β-glycerolphosphate, 0.1 mM PMSF, 1 mM benzamidine, 0.5 mM sodium vanadate, and 1 μM microcystin LR) and centrifuged at 10,000 g for 10 min at 4. The pellet was discarded and the supernatant was aliquoted into microcentrifuge tubes. The protein content was quantified using a detergent-compatible protein assay kit (Bio-Rad), and aliquots of 10 μg were taken individually from each sample, mixed with a one-fifth volume of the sample buffer [0.35 M Tris-Cl, pH 6.8, 10% SDS, 30% glycerol, 9.3% dithiothreitol (DTT), and 0.175 mM bromophenol blue], and then heat-denatured by boiling for 5 min. Separated proteins were transferred to polyvinylidene difluoride membranes (Immobilon-P, Millipore, Bedford, MA) overnight at 4*,* and then incubated with a blocking solution (0.05% Tween 20, 50 mM Tris, pH 8.0, 150 mM NaCl, and 5% powdered non-fat milk) for 1 h. Membranes were then incubated in a 1:500 dilution of monoclonal antibody raised against HSP70 diluted 1:3000 for two hours at room temperature. Similar treatment of the same blot sheet was performed using rabbit polyclonal anti-GAPDH antibody diluted 1:5000 (CSA-400 Stressgen Bioreagents, British Columbia, Canada) after the first antibody treatment had been stripped off. The membrane was incubated with appropriate peroxidase-labeled secondary antibody prepared in PBS-Tween 20. Membranes were then washed and incubated in the goat anti-rabbit secondary antibody (horseradish peroxidase-conjugated goat anti-mouse, Zhongsan Golden Bridge, China) at a 1:5000 dilution at room temperature for 2 h. Primary antibody binding was visualized using an enhanced chemiluminescence kit (Pierce, Rockford, IL) and Hyperfilm-MP (Amersham International, Buckinghamshire, UK).

Immunohistochemical staining was performed on an Alpha Innotech (San Leandro, CA) 8800 image station with FLUORCHEM software using enhanced chemiluminescence (ECL) as the chromagen. The relative intensities of the Western blot membranes were compared using Alpha Ease software.

### Statistical analysis

The data were analyzed by Student’s T-test, with the significance level set at 0.05. A P-value of P > 0.05 but P < 0.1 was taken to indicate a trend. Data are presented as the mean ± standard error.

## Results

### Effect of Arg on liver weight and serum biochemical indices

**Figure 1:**
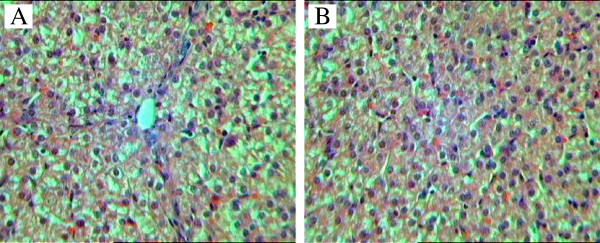
**Photomicrographs of control group (A) and Arg group (B).** Original magnification, 400×.

The liver tended to be heavy in the Arg group (P = 0.071) (Figure 1). Compared to the control piglets, Arg increased serum ALP (P < 0.05), and decreased GOT (P < 0.05), lactic acid (P < 0.05) and COR (P < 0.05). Arg did not affect GLU or amylase (*P* > 0.05).

### Effects of Arg on pathological findings

Histopathological changes such as sinusoidal dilatation, hepatocellular vacuolization and hepatocellular necrosis were present. Moderate cell infiltration was seen in the periportal areas. Histopathologic examination showed increases in lymphocyte and neutrophil infiltration in the central and portal areas in the livers of the control group. In some areas, microvesicular steatosis and hepatocyte degeneration were also observed. Hepatic ischaemia was seen in the control groups. In the Arg group, inflammatory reactions were decreased and other changes were almost absent in liver sections (Figure 1). HAI was lower in the Arg group (*P* < 0.05) (Table 2).

**Table 2:** Effects of Arg on liver weight and serum biochemical indices in weanling piglets (n = 6)

**Items**	**Control**	**Arg**
Initial body weight (kg)	5.9 ± 0.4	5.9 ± 0.4
Final body weight (kg)	6.1 ± 0.3 ^a^	6.6 ± 0.3 ^b^
Feed intake daily (g)	158.3 ± 20.9^a^	180.6 ± 29.3^b^
Liver weight (kg)	140.9 ± 5.0	143.9 ± 5.2
Liver weight/Body weight (%)	2.3 ± 0.1	2.18 ± 0.1
Glucose (mmol/L)	7.7 ± 1.0	6.9 ± 0.6
Glutamic-oxalacetic transaminase (mmol/L)	46.3 ± 10.5^a^	34.5 ± 6.2^b^
Glutamic pyruvic transaminase (mmol/L)	43.0 ± 5.8	45.2 ± 4.9
Alkaline phosphatase (mmol/L)	166.8 ± 50.5^a^	224.2 ± 36.6^b^
Lactic acid (mmol/L)	69.8 ± 12.9^a^	41.2 ± 11.1^b^
Lactate dehydrogenase (U/L)	597.8 ± 69.0	546.5 ± 86.3
Cortisol (μg/L)	88.7 ± 26.1^a^	69.6 ± 22.7^b^

^ab^: means in the same row with different letters differ significantly (*P* < 0.05).

Values are means ± standard error.

### Immunohistochemistry, real-time PCR, and Western blot for HSP70 in liver

**Figure 2:**
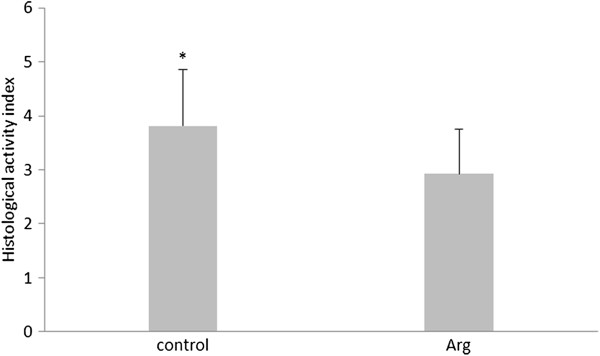
Effect of Arg on histological activity index of piglets.

Immunohistochemistry studies showed different behaviors of HSP70 expression. The immunohistochemical localization of HSP70 in the liver revealed stronger expression in the Arg group (Figure 2).

**Figure 3:**
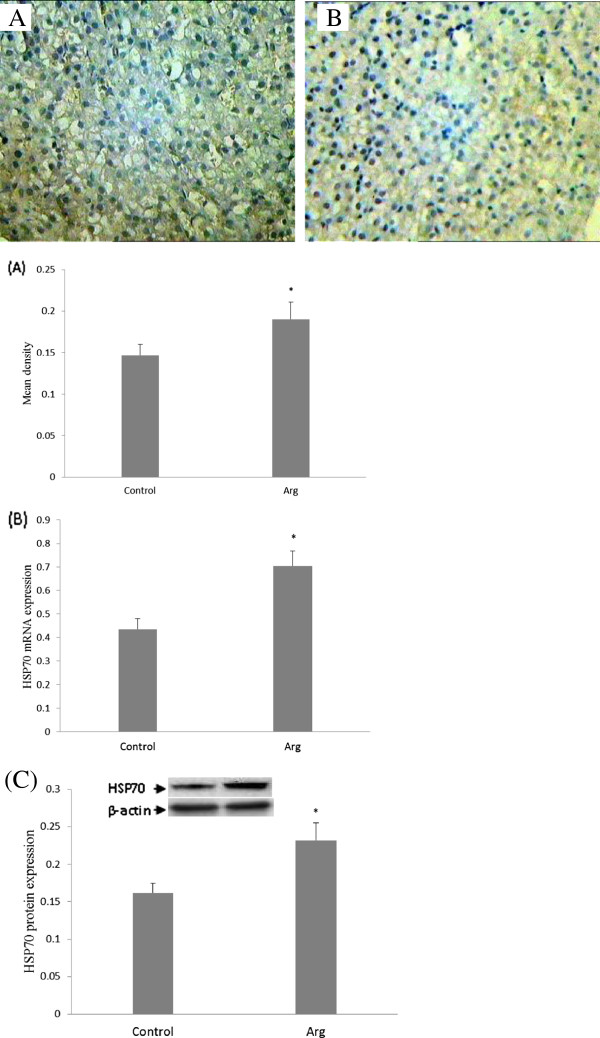
**Immunohistochemistry (A), Real-time PCR (B) and Western blot (C) analysis for HSP70 and β-actin expression in liver tissues.** HSP70 expression showed positive and statistically significant correlation with favorable prognosis. **A** (control group) showed expression of HSP70 staining; **B** stronger expression of HSP70 was observed in the **B** (Arg group). Original magnification 400×. (n = 6). Both HSP70 mRNA and protein were higher in the Arg group (P < 0.05).

Real-time PCR showed that the level of HSP70 mRNA in the liver was higher in the Arg group (*P* < 0.05) (Figure 3). To confirm the results of immunohistochemistry, liver tissues were subjected to Western blot assay. Representative results are shown in Figure 3. The expression of HSP70 in the control group was lower than that in the Arg group (*P* < 0.05).

## Discussion

In rodents and humans, Arg is essential when Arg turnover increases, as in growth, inflammation, or tissue repair, and the dietary supply can become rate-limiting for Arg-metabolizing pathways [7,27]. Indeed, low Arg levels have been documented in weaning-age piglets [21]. It has been reported that, compared to that in newborn pigs, the intestinal synthesis of citrulline and Arg from glutamine and glutamate decreases by 70-73% in 7-d-old suckling pigs, and declines further in 14- to 21-d-old pigs [7].

In the present study, the significant increase in serum COR and the marked decrease in ALP in the control group indicated that weaning caused stress in piglets, and the elevation of AST in serum may indicate that the liver was damaged. Accordingly, the present results indicated that Arg decreased damage to liver tissue after weaning, in comparison to the control group. This is consistent with the results of other studies. For example, the administration of Arg reduces damage to liver tissue after ischaemia injury [13]. In addition, it has been reported that Arg could protect rats with liver cirrhosis from acute ammonia intoxication [28].

HSPs are intracellular chaperones that play a key role in the recovery from stress and are involved in nearly all intracellular compartments. Stress-induced HSP70s function to promote refolding and prevent the aggregation of partially-denatured proteins, as well as tag irreversibly-damaged proteins for proteolysis [29]. Arg deprivation decreases HSP expression, and enhances the cellular susceptibility to apoptosis [30]. HSP70 could be used as a marker for assessing the biological behavior under stress in vivo [31]. It has been reported that the overexpression of liver HSP70, particularly during the summer, may confer differential effects on cell survival by protecting against changes induced by oxidative stress [32]. Our results suggest that Arg increased HSP70 mRNA and protein in the liver. Accordingly, immunohistochemistry studies showed differences in HSP70 expression. Our study demonstrated that the expression of HSP70 in the liver was negatively correlated with an increase in the aggressiveness of the histological type. It has also been reported that HSP70 levels in the liver were significantly increased in heat-stressed mice that had been administered Arg compared with a heat-stress-only group [20]. If we consider the role of HSP70 in RNA-DNA proliferation, these increases may be explained by an increase in cell proliferation and protein synthesis promoted by supplementation with Arg [33,34].

## Conclusions

In summary, the present results indicated that pretreatment with Arg could increase the expression of HSP70 in the liver, which might be able to reduce liver injury and enhance liver health in piglets during weaning.

## Competing interests

The authors declare that they have no competing interest.

## Authors’ contributions

XW and YY designed the study. XW and CX performed the experiments and analyzed the data. XW prepared the manuscript and all of the authors contributed to, read and approved the final manuscript.
